# Structural Basis of the γ-Lactone-Ring Formation in Ascorbic Acid Biosynthesis by the Senescence Marker Protein-30/Gluconolactonase

**DOI:** 10.1371/journal.pone.0053706

**Published:** 2013-01-22

**Authors:** Shingo Aizawa, Miki Senda, Ayaka Harada, Naoki Maruyama, Tetsuo Ishida, Toshiro Aigaki, Akihito Ishigami, Toshiya Senda

**Affiliations:** 1 Cellular Genetics, Graduate School of Science and Engineering, Tokyo Metropolitan University, Hachioji, Tokyo, Japan; 2 Molecular Regulation of Aging, Tokyo Metropolitan Institute of Gerontology, Itabashi-ku, Tokyo, Japan; 3 Biomedicinal Information Research Center (BIRC), National Institute of Advanced Industrial Science and Technology (AIST), Koto-ku, Tokyo, Japan; 4 Research and Development Department, Japan Biological Informatics Consortium (JBIC), Koto-ku, Tokyo, Japan; 5 Materials Science and Engineering, Tokyo Denki University, Adachi-ku, Tokyo, Japan; 6 Department of Biochemistry and Molecular Biology, Shiga University of Medical Science, Ohtsu, Shiga, Japan; National Research Council of Italy, Italy

## Abstract

The senescence marker protein-30 (SMP30), which is also called regucalcin, exhibits gluconolactonase (GNL) activity. Biochemical and biological analyses revealed that SMP30/GNL catalyzes formation of the γ-lactone-ring of l-gulonate in the ascorbic acid biosynthesis pathway. The molecular basis of the γ-lactone formation, however, remains elusive due to the lack of structural information on SMP30/GNL in complex with its substrate. Here, we report the crystal structures of mouse SMP30/GNL and its complex with xylitol, a substrate analogue, and those with 1,5-anhydro-d-glucitol and d-glucose, product analogues. Comparison of the crystal structure of mouse SMP30/GNL with other related enzymes has revealed unique characteristics of mouse SMP30/GNL. First, the substrate-binding pocket of mouse SMP30/GNL is designed to specifically recognize monosaccharide molecules. The divalent metal ion in the active site and polar residues lining the substrate-binding cavity interact with hydroxyl groups of substrate/product analogues. Second, in mouse SMP30/GNL, a lid loop covering the substrate-binding cavity seems to hamper the binding of l-gulonate in an extended (or all-trans) conformation; l-gulonate seems to bind to the active site in a folded conformation. In contrast, the substrate-binding cavities of the other related enzymes are open to the solvent and do not have a cover. This structural feature of mouse SMP30/GNL seems to facilitate the γ-lactone-ring formation.

## Introduction

Senescence marker protein-30 (SMP30) is a 34-kDa protein whose tissue levels in the liver, kidney, and lung decrease with aging [1], [2]. This protein is expressed in several organs but most prominently in the liver and kidney [1], [3]. The amino acid sequences of SMP30 are highly conserved among vertebrates, i.e., 70% to 90% [4]. The SMP30 gene is located on the X chromosome [5]. SMP30 is also designated as regulcalcin [6]. DNA sequence analyses of regucalcin and SMP30 revealed that these proteins are identical. While biological analyses have suggested various homeostatic roles of regucalcin [7], [8], these activities will not be discussed in the present manuscript. In this manuscript, we analyzed only the gluconolactonase activity of SMP30.

**Figure 1:**
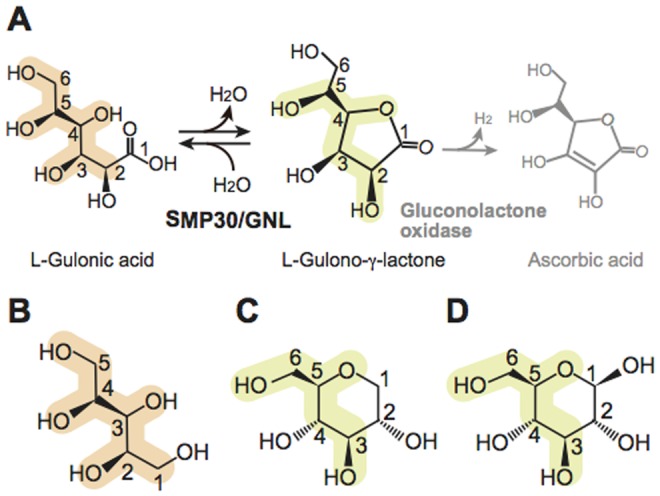
Catalytic reaction of mouse SMP30/GNL. (**A**) The γ-lactone-forming reaction catalyzed by mouse SMP30/GNL. The product of the catalytic reaction of SMP30/GNL is l-gulono-γ-lactone, which is in turn converted to ascorbic acid by gluconolactone oxidase. (**B–D**) Substrate and product analogues used in this study: (**B**) xylitol, (**C**) 1,5-anhydro-d-glucitol (1,5-AG), and (**D**) d-glucose. Corresponding atoms in these molecules are marked in light orange and light yellow.

Biochemical studies suggested that SMP30 is a gluconolactonase (GNL, EC 3.1.1.17) and requires a divalent metal ion such as Zn^2+^ and Mn^2+^ for its gluconolactonase activity [9]. While SMP30 (hereafter SMP30/GNL) is known to catalyze lactone-ring cleavage reactions *in vitro* with various substrates, such as aldonolactones d/l-glucono-δ-lactone, d/l-gulono-γ-lactone, and d/l-galactono-γ-lactone [9], its physiological substrate(s) has remained elusive. The physiological substrate of SMP30/GNL was initially uncovered with SMP30/GNL-knockout (KO) mice by our group. Our biological study demonstrated that the SMP30/GNL-KO mice developed symptoms of scurvy when fed ascorbic acid (vitamin C)-deficient diets, verifying a pivotal role of SMP30/GNL in the ascorbic acid biosynthesis [9]. We therefore concluded that mouse SMP30/GNL catalyzes a lactone-ring formation–a conversion of l-gulonate to l-gulono-γ-lactone–in the ascorbic acid biosynthesis pathway (**Figure 1A**) [9]. This was the first report of a physiological function of SMP30/GNL. It seems reasonable to consider that SMP30/GNL is involved in the ascorbic acid biosynthesis in many animals. However, since humans, monkeys, and guinea pigs are unable to synthesize ascorbic acid *in vivo* due to a loss-of-function genetic mutation of the gluconolactone oxidase, which is the last enzyme of the ascorbic acid biosynthesis that converts l-gulono-γ-lactone to l-ascorbic acid [9], [10], SMP30/GNL may have another physiological function, at least in these animal cells.

Recently, the crystal structure of human SMP30/GNL was determined at 1.4 Å resolution [11]. The crystal structure showed that human SMP30/GNL adopts a six-bladed β-propeller fold [12], which is similar to those of squid diisopropyl-fluorophosphatase (DFPase) [13], drug resistance protein 35 (Drp35) [14], and paraoxonase (PON) [15]. These proteins show an amino acid sequence similarity with human SMP30/GNL. A cavity inside the β-propeller structure of human SMP30/GNL contains a divalent metal ion (Ca^2+^ and Zn^2+^) present in the crystallization solution and the artificial mother liquor used for crystal soaking. The metal ion coordinates of three amino acid residues, Glu18, Asn154, and Asp204. Since SMP30/GNL requires a metal ion such as Zn^2+^ and Mn^2+^ for its enzyme activity, the active site of SMP30/GNL must be located inside the cavity. A site-directed mutagenesis study of human SMP30/GNL revealed that the bound metal ion and Asn103 are critical to the catalytic reaction of human SMP30/GNL. Although the crystal structure of human SMP30/GNL was determined, its physiological functions remain elusive.

In the ascorbic acid biosynthesis pathway, the ring structure of d-glucuronate is cleaved to generate l-gulonate, and then a γ-lactone ring, which is a basic structural framework of ascorbic acid, is formed from l-gulonate by SMP30/GNL [9]. While the formation of the γ-lactone ring is a critical step in the synthesis of ascorbic acid, the catalytic mechanism of the γ-lactone-ring formation remains elusive due to the lack of structural information of the substrate complex of SMP30/GNL. Furthermore, while some of the related enzyme structures have been determined [13]–[15], no structural information has been obtained for substrate complexes relevant to the GNL activity. Here, we report the crystal structures of mouse SMP30/GNL not only in the substrate-free form but also in the substrate/product-analogue complex forms (**Figure 1B, C, D**). These structural analyses revealed the molecular basis of the γ-lactone-ring formation in the ascorbic acid biosynthesis.

## Materials and Methods

### Cloning, expression, and purification of mouse and human SMP30/GNL

The entire coding sequences of mouse and human SMP30/GNL (GenBank U28937, nt 73 to 972 and GenBank D31815, nt 350 to 1249, respectively) [5], [16] were amplified by polymerase chain reaction (PCR) using the following primers: mouse SMP30/GNL 5′-forward primer 5′-GCGCGGAATTCCATATGTCTTCCATCAAAGTTGAATG-3′ (37 mer), mouse SMP30/GNL 3′-reverse primer 5′-ATATAAGCTTCTCGAGTCACCCTGCATAGGAATAT G-3′ (36 mer), human SMP30/GNL 5′-forward primer 5′-GCGCGGAATTCCATATGTCTTCCATTAAGATTGAGTG-3′ (37 mer) and human SMP30/GNL 3′-reverse primer 5′-ATATAAGCTTCTCGAGTCATCCCGCATAGGAGTAGG-3′ (36 mer). SMP30/GNL PCR products were subcloned into the pET-21b(+) expression vector (Novagen), resulting in pET-21b(+)/mouse SMP30/GNL and pET-21b(+)/human SMP30/GNL. *Escherichia coli* BL21(DE3) and BL21(DE3)-Rossetta2/pLysS were transformed with pET-21b(+)/mouse SMP30/GNL and pET-21b(+)/human SMP30/GNL, respectively. *E. coli* BL21(DE3) [pET21b(+)/mouse SMP30/GNL] were grown in LB medium containing 1% (v/v) glycerol, 1% (w/v) glucose, and 50 µg/ml carbenicillin. *E. coli* BL21(DE3)-Rossetta2/pLysS [pET21b(+)/human SMP30/GNL] were grown in the same medium supplemented with 30 µg/ml chloramphenicol. The expression of SMP30/GNL was induced at an OD_600_ of 0.4–0.6 with 0.5 mM isopropyl-β-d-thiogalactopyranoside (IPTG), and the cells were cultured for 20 hr before harvesting. Harvested cells were suspended in a lysis buffer (20 mM Tris-HCl pH 7.5, 5% glycerol, 5 mM β-mercaptoethanol) and disrupted by sonication. The cell lysate was centrifuged at 21,000 *g* for 30 min at 4°C. The resultant supernatant was used as a cell extract.

Mouse SMP30/GNL was purified as follows. The cell extract was applied to a TOYOPEARL DEAE-650M column (TOSOH, 50 ml) equilibrated with the lysis buffer. Flow-through fractions containing mouse SMP30/GNL were collected and dialyzed against buffer A (20 mM Tris-HCl pH 9.0, 5% glycerol, 5 mM β-mercaptoethanol). The sample was then loaded onto a TOYOPEARL SuperQ-650M column (TOSOH, 50 ml) pre-equilibrated with buffer A. Mouse SMP30/GNL was eluted by a gradient from 0% to 100% of buffer B (20 mM Tris-HCl pH 9.0, 100 mM NaCl, 5% glycerol, 5 mM β-mercaptoethanol). The buffer of the eluted sample was exchanged with buffer C (20 mM Tris-HCl pH 8.0, 1.0 M ammonium sulfate) by ultrafiltration with an Amicon Ultra-15 centrifugal filter unit (MWCO 10,000) (Millipore). Then, the sample was applied to a Phenyl-Sepharose CL-4B column (SIGMA-ALDRICH, 50 ml) pre-equilibrated with buffer C. Mouse SMP30/GNL was eluted by a gradient from 0% to 100% of buffer D (20 mM Tris-HCl pH 8.0). Eluted fractions were concentrated using an Amicon Ultra-15 centrifugal filter unit (MWCO 10,000) (Millipore) and further purified using a HiLoad 16/60 Superdex 200 pg column (GE Healthcare, 120 ml) pre-equilibrated with buffer E (20 mM Tris-HCl pH 8.0, 300 mM NaCl, 5% glycerol, 5 mM β-mercaptoethanol). Fractions containing mouse SMP30/GNL were collected and dialyzed against buffer D.

Human SMP30/GNL was purified as follows. The cell extract was fractionated by ammonium sulfate precipitation (45%–70% saturation), dissolved in the lysis buffer, and dialyzed overnight at 4°C against the lysis buffer. The dialyzed sample was centrifuged at 21,000 *g* for 30 min, and the supernatant was applied to a TOYOPEARL DEAE-650M column (TOSOH, 500 ml) equilibrated with the lysis buffer. Flow-through fractions containing human SMP30/GNL were collected and the buffer was exchanged with buffer C by ultrafiltration using an Amicon Ultra-15 centrifugal filter unit (MWCO 10,000) (Millipore). The obtained sample was applied to a Phenyl-Sepharose CL-4B column (SIGMA-ALDRICH, 50 ml) pre-equilibrated with buffer C. Human SMP30/GNL was eluted by a gradient from 0% to 100% of buffer D. The eluted fractions were concentrated using an Amicon Ultra-15 centrifugal filter unit (MWCO 10,000) (Millipore) and further purified using a HiLoad 16/60 Superdex 200 pg column (GE Healthcare, 120 ml) pre-equilibrated with buffer E. The eluted fractions were dialyzed against buffer D.

Purified proteins were analyzed by sodium dodecyl sulfate-polyacrylamide gel electrophoresis (SDS-PAGE) with Coomassie brilliant blue (CBB) stain (**Figures S1A, B**).

### Measurement of the GNL activity

The GNL activity of the purified sample was measured by monitoring the pH change caused by free acid formation from d-glucono-δ-lactone [9], [17]. The pH change was measured using the pH indicator *p*-nitrophenol. The reaction mixture for the GNL-activity measurement contained 10 mM d-glucono-δ-lactone, 10 mM PIPES (pH 6.4), 0.25 mM *p*-nitrophenol, 75 µM ZnCl_2_, and the enzyme in a total volume of 1 ml. The reaction was monitored with absorbance at 405 nm. The protein concentrations of mouse and human SMP30/GNL were usually measured by the Bradford method using bovine serum albumin (SIGMA-ALDRICH) as the standard. The obtained concentrations of mouse and human SMP30/GNL by the Bradford method were nearly the same as those measured by Abs_280_ with theoretical molar extinction coefficients of 1.507 and 1.473 M^−1^ cm^−1^ for mouse and human SMP30/GNL, respectively.

### Measurement of divalent metal ions

The amounts of Zn^2+^, Mn^2+^, Mg^2+^, and Ca^2+^ ions in the purified mouse and human SMP30/GNL were analyzed using inductively coupled plasma mass spectrometry (ICP-MS). Samples analyzed by ICP-MS were (a) purified mouse SMP30/GNL (1.2 mg/ml), (b) the dialyzed sample of mouse SMP30/GNL (sample (a)) against 20 mM Tris-HCl pH 8.0 (0.5 mg/ml), and (c) the dialyzed sample of mouse SMP30/GNL (sample (a)) against 20 mM Tris-HCl pH 8.0, 1 mM 1,10-phenanthroline, 1 mM Tiron, 1 mM EDTA, and 1 mM EGTA (0.8 mg/ml), (d) purified human SMP30/GNL (1.2 mg/ml), (e) the dialyzed sample of human SMP30/GNL (sample (d)) against 20 mM Tris-HCl pH 8.0 (1.2 mg/ml), and (f) the dialyzed sample of human SMP30/GNL (sample (d)) against 20 mM Tris-HCl pH 8.0, 1 mM 1,10-phenanthroline, 1 mM Tiron, 1 mM EDTA, and 1 mM EGTA (1.2 mg/ml).

Samples (1 ml) were analyzed by Sumika Chemical Analysis Service, Ltd. (Osaka, Japan).

### Crystallization

The purified mouse SMP30/GNL was concentrated to 13–15 mg/ml using an Amicon Ultra-0.5 centrifugal filter unit (MWCO 10,000) (Millipore). The initial screening of crystallization conditions was attempted with a Crystal Screen I (Hampton Research) using the hanging-drop vapor diffusion method at 20°C. Typically, a 2 µl droplet was prepared by mixing 1 µl of the protein solution and 1 µl of the reservoir solution. Initial crystals were obtained from a droplet of reagent #4 of Crystal Screen I (2.0 M ammonium sulfate and 100 mM Tris-HCl, pH 8.5). Optimization of the crystallization conditions gave tetragonal bipyramid crystals with a reservoir solution of 1.6 M ammonium sulfate and 100 mM Tris-HCl, pH 8.5. The crystals grew within a few days to the maximum dimensions of approximately 0.2×0.14×0.14 mm^3^ (**Figure S1C**).

Human SMP30/GNL was crystallized using the conditions reported earlier by Chakraborti *et al.* [11]. Human SMP30/GNL was concentrated to 10–12 mg/ml by using an Amicon Ultra-0.5 centrifugal filter unit (MWCO 10,000) (Millipore) and crystallized by the sitting-drop vapor diffusion method at 20°C. The reported crystallization conditions were further optimized and a reservoir solution of 28% PEG6000, 100 mM Tris-HCl pH 7.5, and 5 mM CaCl_2_ gave the best crystal with approximate dimensions of 0.3×0.35×0.02 mm^3^ (**Figure S1D**).

### X-ray data collection and structure determination

Diffraction data of mouse and human SMP30/GNL were collected at 95K with an ADSC CCD detector using synchrotron radiation at BL-5A, BL-17A, and PF-AR NE-3A of Photon Factory (PF) in KEK (Tsukuba Japan). The mouse SMP30/GNL crystals were cryo-protected with 100% paratone-N. The human SMP30/GNL crystals were cryo-protected with 100 mM Tris-HCl pH 7.5, 28% PEG6000, 30% PEG400, and 5 mM CaCl_2_ [11].

A cryo-protected crystal was mounted on a cryoloop (Hampton Research) and flash-cooled in an N_2_ flow at 95 K. The diffraction data were processed and scaled using the programs XDS and XSCALE, respectively [18]. The crystal structure of human SMP30/GNL was determined by the molecular replacement (MR) method with the program MOLREP [19] in the CCP4 program suite [20] using the earlier determined crystal structure of human SMP30/GNL (PDB ID: 3G4E) [11] as a search model. Then, the structure of mouse SMP30/GNL was determined by the MR method using the structure of human SMP30/GNL. The crystal structures of mouse and human SMP30/GNL were refined using the program phenix.refine [21]. Molecular models were built using the program COOT [22].

Crystals of mouse/human SMP30/GNL in complex with 1,5-Anhydro-d-glucitol (1,5-AG) were prepared by the soaking method. Mouse SMP30/GNL crystals were soaked for 6 hr in the artificial mother liquor for the mouse SMP30/GNL crystal (1.8 M ammonium sulfate, 100 mM Tris-HCl pH 8.5) supplemented with 500 mM 1,5-AG (Wako). The resultant crystals were cryoprotected by soaking in 100% paratone-N for 30 sec. Human SMP30/GNL crystals were soaked for 8 hr in 100 mM Tris-HCl pH 7.5, 28% PEG6000, and 5 mM CaCl_2_ supplemented with 500 mM 1,5-AG (Wako) and cryoprotected by soaking in 100 mM Tris-HCl pH 7.5, 28% PEG6000, 30% PEG400, 5 mM CaCl_2_, and 500 mM 1,5-AG for 30 sec.

Crystals of the mouse SMP30/GNL-d-glucose complex were prepared by soaking in the artificial mother liquor for mouse SMP30/GNL containing 28% (w/v) d-glucose for 30 sec. Crystals of the mouse SMP30/GNL-xylitol complex were prepared by soaking in the artificial mother liquor containing 50% (w/v) xylitol for 2 min. These crystals could properly be frozen by a N_2_ stream at 95K without further cryo-protection. Diffraction data collection and structure determination of the substrate/product-analogue complexes were performed as described above.

The geometry of the refined crystal structures were assessed by the program PROCHECK [23] in the CCP4 program suite [20]. All molecular graphics in this manuscript were prepared by the program Pymol [24]. Structure comparisons of mouse and human SMP30/GNL were performed using the program Lsqkab in the CCP4 program suite [20]. Simulated annealing omit maps (SA-omit maps) were calculated by phenix.refine [21].

### Database search

A homology search of tertiary structures was performed by the program MATRAS (http://strcomp.protein.osaka-u.ac.jp/matras/) [25] using the crystal structure of mouse SMP30/GNL as a template. All coordinates in the PDB were examined by this search.

### Molecular weight determination

The molecular weights of mouse and human SMP30/GNL were determined by low-angle laser light scattering measurement combined with gel chromatography using a TSKgel SuperSW2000 column (φ 4.6 mm×300 mm) according to the previously described method [26] with slight modification. The elution buffer was 50 mM HEPES, pH 7.5, ionic strength 0.15 mol/L, and the flow rate was 0.2 ml/min. The molecular weight standards used were bovine pancreatic ribonuclease A (*M*_r_ = 13,700), chicken egg albumin monomer (*M*_r_ = 45,000), and bovine albumin monomer (*M*_r_ = 66,300); these proteins were purchased from SIGMA-ALDRICH.

## Results

### Purification of recombinant mouse and human SMP30/GNL

Recombinant mouse and human SMP30/GNL were successfully purified by sequential column chromatography. Each protein yielded a single major band of approximately 34 kDa on SDS-PAGE followed by CBB staining (**Figures S1A, B**). The purity of each protein was estimated as approximately 95%. The GNL activities of the purified mouse and human SMP30/GNL at 25°C were (1.30±0.03)×10^3^ µmol/min/mg and 799±60 µmol/min/mg, respectively. To exclude the possibility that the GNL activity was ascribable to the 5% protein contaminants, we analyzed the GNL activity of a crude extract of *E. coli* cells that harbors no SMP30/GNL gene. Crude extract from *E. coli* without the SMP30/GNL gene showed no detectable GNL activity (**Table S1**). While the crude extract supplemented with purified SMP30/GNL showed the GNL activity, the GNL activity was not affected by changing the total amount of the crude extract in the reaction mixture (**Table S2**). This result showed that SMP30/GNL is unlikely to function as an activator of the GNL activity in the contaminants. It is of note that an earlier paper suggested that the active site for the GNL activity of SMP30/GNL is located inside the β-propeller structure on the basis of mutational analysis [11]. We thus concluded that SMP30/GNL has the GNL activity.

### Analysis of divalent metal ions in mouse and human SMP30/GNL

Initially, SMP30/GNL (regucalcin) that was purified from rat liver was considered to be a Ca^2+^-binding protein [6]. Recent biochemical analysis, however, suggested that SMP30/GNL also binds Zn^2+^, Mn^2+^, and Mg^2+^ [11]. Chakraborti *et al.* showed that the addition of the Ca^2+^ ion to the enzyme weakly activated the GNL activity of human SMP30/GNL and calculated the *K*_d_ value for the Ca^2+^ ion on the basis of the enzymatic data [11]. Their biochemical analysis showed that Zn^2+^, Mn^2+^, and Mg^2+^ also bound to human SMP30/GNL and activated its GNL activity. In order to analyze divalent metal ions in the recombinant SMP30/GNL, purified recombinant mouse and human SMP30/GNL were analyzed by ICP-MS (**Table S3**). While Zn^2+^, Mn^2+^, Mg^2+^, and Ca^2+^ were contained in the purified mouse and human SMP30/GNL, approximately half of the total amount of purified SMP30/GNL contained the Ca^2+^ ion. After dialysis, however, most of the bound divalent metal ions were dissociated from mouse and human SMP30/GNL even without chelating reagents (**Table S3**).

### Structure determination of mouse and human SMP30/GNL

The crystal structures of mouse and human SMP30/GNL in the substrate-free form were determined at 1.95 Å and 1.50 Å resolutions, respectively (**Tables 1, 2**). In the course of the crystallographic refinement, a strong electron density was found inside the β-propeller structure in both mouse and human SMP30/GNL. Earlier structure analysis of relevant molecules [11], [13]–[15] suggested that the strong density represents a divalent metal ion. The ICP-MS analysis suggested that the divalent metal ion bound to SMP30/GNL molecules in the crystal showed heterogeneity; the SMP30/GNL molecules bound Mn^2+^, Zn^2+^, Mg^2+^, or Ca^2+^ in the crystal (**Table S3**). Therefore, we use the term *divalent metal ion* to refer to the bound metal ion in the active site of SMP30/GNL in this manuscript. Since Ca^2+^ was the most abundant metal ion in the purified sample (**Table S3**), a Ca^2+^ ion was placed at the metal site for the crystallographic refinement.

**Table 1:** Crystallographic data summary of mouse SMP30/GNL.

Species	Mouse	Mouse	Mouse	Mouse
Bound compound	Substrate free	1,5-AG	Glucose	Xylitol
***Data collection***				
Light source	PF BL-17A	PF BL-17A	PF-AR NE-3A	PF BL-17A
Space group	*P*3_1_2 1	*P*3_1_2 1	*P*3_1_2 1	*P*3_1_2 1
Cell dimensions (Å)				
*a*	102.68,	102.59,	101.86	101.97
*b*	102.68,	102.59,	101.86	101.97
*c*	147.82,	149.71,	146.83	147.55
*α, β, γ*(°)	90, 90, 120	90, 90, 120	90, 90, 120	90, 90, 120
Resolution (Å)	76.2–1.95	76.4–1.70	75.62–2.00	56.62–1.85
Highest resolution shell	2.06–1.95	1.79–1.70	2.11–2.00	1.95–1.85
*R*_merge_	0.050 (0.445)	0.042 (0.494)	0.076 (0.643)	0.046 (0.475)
*I*/σ	31.34 (6.61)	37.56 (6.75)	23.0 (3.96)	34.07 (5.97)
Completeness (%)	100.0 (100.0)	99.9 (100.0)	99.9 (100.0)	99.9 (100.0)
Redundancy	10.9 (11.1)	10.8 (11.0)	7.3 (7.4)	10.8 (11.0)
***Refinement***				
Resolution (Å)	51.34–1.95	49.90–1.70	75.62–2.00	56.62–1.85
No. reflections	66221	100500	60052	76106
*R*_work_	0.1852	0.1705	0.1759	0.1657
*R*_free_	0.2153	0.1895	0.2076	0.1845
No. atoms				
Protein	4638	4724	4680	4688
Ligand/ion	0/67	101/67	96/32	20/26
Water	373	470	334	403
*B*-factors (Å^2^)				
Protein	31.6	22.4	31.3	28.0
Ligand/ion	-/47.3	32.6/36.6	48.9/46.2	41.0/43.1
Water	36.1	32.0	35.9	34.4
Rms deviations				
Bond lengths (Å)	0.007	0.007	0.014	0.006
Bond angles (deg)	1.169	1.166	1.228	1.141
PDB ID	4GN7	4GN8	4GN9	4GNA

* Values for the highest resolution shell are shown in parenthesis.

**Table 2:** Crystallographic data summary of human SMP30/GNL.

Species	Human	Human
Bound compound	Substrate free	1,5-AG
***Data collection***		
Light source	PF BL-5A	PF BL-17A
Space group	*P*2_1_	*P*2_1_
Cell dimensions (Å)		
*a*	64.50,	64.41,
*b*	50.79,	50.20,
*c*	86.56	86.57,
*α, β, γ*(°)	90, 100.24, 90	90, 99.65, 90
Resolution (Å)	20.0–1.50	50.0–1.75
Highest resolution shell	1.58–1.50	1.81–1.75
*R*_merge_	0.045 (0.530)	0.058 (0.375)
*I*/σ	24.09 (3.59)	19.48 (2.24)
Completeness (%)	98.3 (96.0)	99.3 (97.2)
Redundancy	5.3 (4.9)	4.6 (3.8)
***Refinement***		
Resolution (Å)	19.53–1.50	47.29–1.75
No. reflections	86999	54737
*R*_work_	0.1893	0.1951
*R*_free_	0.2181	0.2300
No. atoms		
Protein	4613	4535
Ligand/ion	0/3	55/2
Water	391	159
*B*-factors (Å^2^)		
Protein	15.6	24.485
Ligand/ion	-/13.4	30.249/20.420
Water	24.7	26.268
Rms deviations		
Bond lengths (Å)	0.007	0.011
Bond angles (deg)	1.144	1.139
PDB ID	4GNB	4GNC

* Values for the highest resolution shell are shown in parenthesis.

The asymmetric unit of the mouse and human SMP30/GNL contained two SMP30/GNL molecules. However, molecular weight analysis with static light scattering revealed that mouse and human SMP30/GNL exist as a monomer in solution (**Figure S2**). The estimated molecular weights of mouse and human SMP30/GNL in solution were (3.12±0.10)×10^4^ Da and (3.15±0.16)×10^4^ Da, respectively. In addition to the substrate-free form, crystal structures of substrate/product-analogue binding forms were determined (****Tables 1, 2**,** **Figures 1B, C, D**). We found that mouse SMP30/GNL can bind 1,5-AG, glucose, and xylitol to the substrate-binding cavity. The geometries of the refined crystal structures were analyzed by the program PROCHECK [23], and the results showed that no residues of mouse and human SMP30/GNL have φ–ψ angles in the disallowed region of the Ramachandran plot (**Figure S3**).

### Overall structure of mouse SMP30/GNL

**Figure 2:**
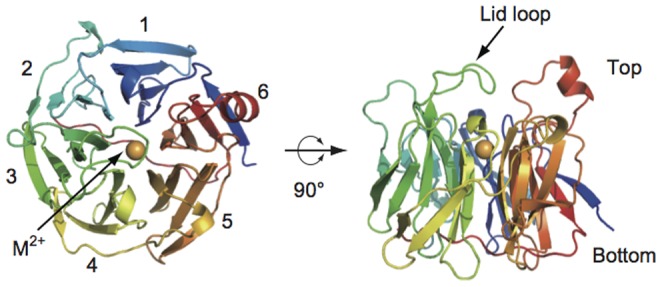
Overall structure of mouse SMP30/GNL. The structure is shown as a rainbow colored cartoon with N-terminus = blue and C-terminus = red. The divalent metal ion (labeled as M^2+^) located at the center of the structure is shown as an orange sphere.

**Figure 3:**
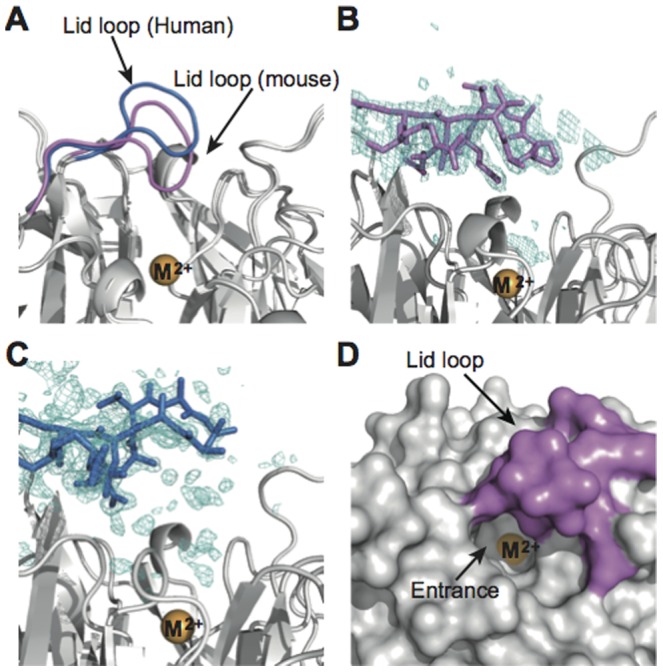
Structural comparison of the lid loops of mouse and human SMP30/GNL. (**A**) Lid loops of mouse and human SMP30/GNL in the substrate free form are shown in purple and blue, respectively. The divalent metal ion (labeled as M^2+^) is shown in orange. (**B, C**) SA-omit maps (mFo-DFc maps) for the lid loop residues in mouse (**B**) and human (**C**) SMP30/GNL. The contour levels of the SA-omit maps are 3.0 σ and 2.0 σ for panels B and C, respectively. (**D**) Surface representation of mouse SMP30/GNL around the lid loop. The entrance for the substrate-binding cavity is indicated by an arrow. Residues in the lid loop are shown in purple.

The overall structure of mouse SMP30/GNL was essentially the same as that of human SMP30/GNL [11]. Least-squares fitting of mouse and human SMP30/GNL with corresponding 297 Cα atoms gave a root-mean-square (rms) deviation of approximately 0.6 Å (**Table S4**). SMP30/GNL adopts a β-propeller structure, which is composed of six β-sheets each of which is formed with four β-strands (**Figure 2**). The superposition of mouse and human SMP30/GNL revealed that residues 120–129, which are located in a loop region connecting two β-strands, have different conformations between them (**Figures 3A, C, C**). These residues are located at the top of the SMP30/GNL molecule (**Figure 2**), serving as a lid over the substrate-binding cavity of the SMP30/GNL molecule. Packing analysis and structure-based sequence comparison of the mouse and human SMP30/GNL could not give a reasonable explanation for the conformational difference of the lid loops between these two proteins. Notably, the conformation differences are retained in the substrate/product-analogue binding forms (**Figure S4**).

**Figure 4:**
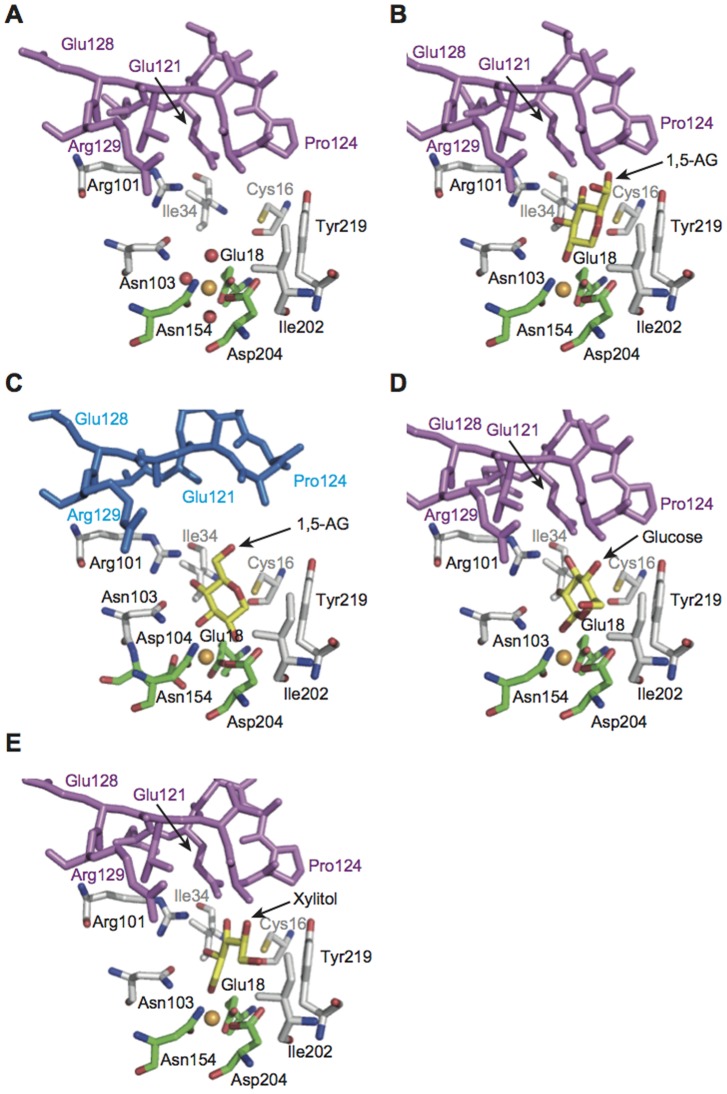
Active site structures of mouse and human SMP30/GNL. (**A**) Mouse SMP30/GNL in the substrate-free form, (**B**) the mouse SMP30/GNL–1,5-AG complex, (**C**) the human SMP30/GNL–1,5-AG complex, (**D**) the mouse SMP30/GNL–d-glucose complex, and (**E**) the mouse SMP30/GNL–xylitol complex. Lid loop residues of mouse SMP30/GNL and human SMP30/GNL are shown in purple and blue, respectively. Carbon atoms of ligand residues for the divalent metal ion (orange sphere) and those for substrate/product analogues are shown in green and yellow, respectively. Other carbon atoms are shown in white.

At the side of the lid loop, there is an entrance for the substrate-binding cavity (**Figure 3D**). The entrance of the cavity is formed by Arg15, Pro35, Pro57, Arg101, Glu120, Glu121, Thr122, Ala123, Pro124, Asp265, and Gly266 in mouse SMP30/GNL. The substrate-binding cavity is large enough to accommodate substrates of SMP30/GNL, and the divalent metal ion is located at the bottom of the cavity. In the substrate-free form, the divalent metal ion coordinates three amino acid residues, Glu18, Asn154, and Asp204 (**Figure 4A** **, Table S5**). In addition, the divalent metal ion coordinates three water molecules. The coordination geometry of the divalent metal ion was quite similar to that of human SMP30/GNL [11].

Amino acid sequence analysis revealed that mouse SMP30/GNL showed 89% amino acid identity with human SMP30/GNL (**Figure S5**). Notably, the amino acid residues lining the substrate-binding cavity were completely conserved. The non-conserved amino acid residues were exclusively located at the outer surface of the SMP30/GNL molecule. Most of the non-conserved amino acid residues were exposed to solvent and located at loop regions connecting secondary structural elements.

### Crystal structures of the mouse and human SMP30/GNL–1,5-AG complexes

Crystal structures of mouse and human SMP30/GNL in complex with 1,5-AG showed that 1,5-AG was bound to the substrate-binding cavity. In mouse SMP30/GNL, the divalent metal ion coordinates the hydroxyl group at the C2 position (the OH(2) group) (**Figure 4B** **, Figure S6A**). The other hydroxyl groups form hydrogen bonds with residues inside the substrate-binding cavity. Arg101, Asn103, and Glu121, which are located at the same side of the substrate-binding cavity, seem to play a critical role in the interaction with 1,5-AG. The OH(3) group of 1,5-AG forms hydrogen bonds with Arg101 and Asn103. The OH(4) and OH(6) groups of 1,5-AG interact with Glu121 in the lid loop (**Figure 4B**).

Interestingly, the binding mode of 1,5-AG in human SMP30/GNL was significantly different from that in mouse SMP30/GNL. The divalent metal ion in the active site coordinates two hydroxyl groups, OH(2) and OH(3), of 1,5-AG with coordination distances of approximately 2.4 Å (**Figure 4C** **, Figure S6B, Table S6**). Arg101 and Asn103 form hydrogen bonds with hydroxyl groups of 1,5-AG as observed in mouse SMP30/GNL. Glu121 on the lid loop, however, does not interact with 1,5-AG in human SMP30/GNL, because Glu121 is located out of the range of interaction (**Figure 4C**); no significant conformational changes were observed in the lid loop upon 1,5-AG binding. Furthermore, the residues in the lid loop of human SMP30/GNL exhibited significantly higher B-factors than other residues in both the substrate-free and 1,5-AG complex forms (**Figure S7A**). Accordingly, electron density for the side chain of Glu121 was invisible. On the other hand, the residues in the lid loop of mouse SMP30/GNL did not seem to be highly mobile (**Figure S7B**), and electron densities for these residues were clearly observed in the simulated-annealing (SA) omit maps (**Figures S4B, E, and G**).

The coordination sphere of the 1,5-AG complex also showed a difference between mouse and human SMP30/GNL. While the three protein ligands for the divalent metal ion showed no significant conformational changes, the side chain of Asp104, which is located at the opposite side of bound 1,5-AG with respect to the divalent metal ion, underwent a conformational change upon 1,5-AG binding in human SMP30/GNL, resulting in an interaction between the side-chain of Asp104 and the divalent metal ion (**Figure S6B**). However, no conformational change of Asp104 was observed in mouse SMP30/GNL upon 1,5-AG binding.

### Binding geometries of glucose and xylitol in mouse SMP30/GNL

In addition to 1,5-AG, interactions between mouse SMP30/GNL and other substrate/product analogue molecules were examined. d-Glucose exhibited a binding mode similar to that of 1,5-AG (**Figure 4D** **, Figure S6C, Table S7**). One hydroxyl group of d-glucose, OH(1), was coordinated to the divalent metal ion. The hydroxyl groups OH(2), OH(3), and OH(4) faced one side of the inner surface of the substrate-binding cavity, as observed in the mouse SMP30/GNL–1,5-AG complex, and formed hydrogen bonds with Arg101, Asn103, and Glu121. Residues on the opposite inner surface of the substrate-binding cavity (Cys16, Ile202, and Tyr219), however, formed no hydrogen bonds with the bound glucose.

Xylitol also binds to the substrate-binding cavity of mouse SMP30/GNL (**Figure 4E** **, Figure S6D**). As observed in other complexes, the OH(1) group is coordinated to the divalent metal ion (**Table S7**). The OH(2), OH(3), and OH(4) groups form hydrogen bonds with Arg101, Asn103, and Glu121. In addition, since Pro124 and Ala125 of the lid loop are located just above the bound xylitol, the xylitol cannot adopt an extended (all trans) conformation; instead, the bound xylitol has a folded conformation (**Figure 4E** **, Figure S6D**).

### Comparison with other related enzymes

A structural homology search using mouse SMP30/GNL by the program MATRAS [25] identified 234 structurally homologous proteins. Of the 234 structures, only four proteins (PDB ID: 1W2T, 3AKH, 3PIJ, 3R4Z) were structurally determined in complex with a monosaccharide (or poly-saccharide) molecule. These molecules are located inside the cavity of the β-propeller fold. However, these four proteins adopt a five-bladed β-propeller fold. Furthermore, the activities of these enzymes are distinct from mouse SMP30/GNL; they are carbohydrate chain-hydrolyzing enzymes. These results indicated that our crystal structures of mouse SMP30/GNL in complex with substrate/product-analogues are the first example showing the substrate-enzyme interactions of GNL enzymes.

The crystal structure of mouse SMP30/GNL was compared with other related enzymes, human SMP30/GNL [11], DFPase [13], Drp35 [14], PON [15], and a putative GNL (PDB ID: 3E5Z) in PDB. The overall folds of these proteins were essentially the same, i.e., they adopted a six-bladed β-propeller structure. The secondary structure matching (SSM) fitting using the program Superpose [27] of these structures with Cα atoms gave rms deviation values ranging from 1.8 Å to 2.9 Å (**Table S8**). These comparisons revealed unique characteristics of SMP30/GNL, particularly in the case of mouse SMP30/GNL. First, the coordination geometry of the divalent metal ion was different between SMP30/GNL and other related enzymes. In DFPase, Drp35, PON, and the putative GNL (PDB ID: 3E5Z), the divalent metal ion in the active site coordinates four protein ligands, which correspond to Glu18, Asn154, Asp204, and Asn103 in mouse/human SMP30/GNL. On the other hand, three residues, Glu18, Asn154, and Asp204, are coordinated to the divalent metal ion in mouse/human SMP30/GNL (**Figure 4A**). Although Asn103 is located close to the divalent metal ion in SMP30/GNL, Asn103 is not coordinated to the divalent metal ion but interacts with a bound substrate/product-analogue molecule (**Figure 4**). Second, the linear arrangement of the polar amino acid residues Arg101, Asn103, and Glu121 in the substrate-binding pocket was found only in the mouse SMP30/GNL. These residues interact with 1,5-AG (**Figure 4B**). Due to the structural difference between mouse and human SMP30/GNL in the lid loop, Glu121 in human SMP30/GNL could not be involved in the interaction with bound 1,5-AG. While the other related enzymes have the corresponding residue for Asn103, no corresponding residues for Arg101 and Glu121 were found in their active sites.

**Figure 5:**
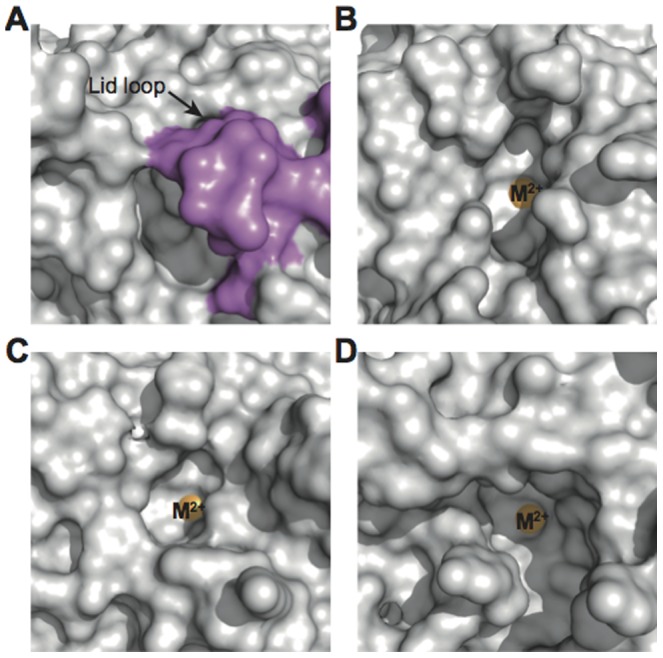
Top view of the substrate-binding cavity. Surface representation of (**A**) mouse SMP30/GNL, (**B**) DFPase, (**C**) Drp35, and (**D**) PON. Residues in the lid loop of mouse SMP30/GNL and the divalent metal ions (labeled as M^2+^) are shown in purple and orange, respectively. Structures of DFPase, Drp35, and PON are superposed onto mouse SMP30/GNL by the SSM fitting using the program Superpose [27] in the CCP4 program suite [20], and all molecules are viewed from the same direction.

Interestingly, mouse SMP30/GNL has another unique characteristic. While there is an entrance hole of the substrate-binding cavity at the side of the lid loop (**Figure 3D**), the lid loop of mouse SMP30/GNL covers the top of the substrate-binding cavity (**Figure 3D**, **Figure 5A**). On the other hand, the substrate-binding cavity for DFPase, Drp35, PON, and the putative GNL are open to the solvent, and no lid structure was found (**Figures 5B, C, D**). The lid loop is known to be a unique structural feature of mouse and human SMP30/GNL [11]. However, since the lid loop of human SMP30/GNL shows a structural difference from that of mouse SMP30/GNL (**Figure 3A** **, Figure S4A**), the space in the substrate-binding cavity of human SMP30/GNL is larger than that of mouse SMP30/GNL, resulting in the difference in the 1,5-AG-binding mode (**Figures 4B, C**).

Notably, it has been reported that SMP30/GNL exhibits a DFPase activity [28]–[30], although it has only approximately 20% sequence identify with squid DFPase. A biochemical study on squid DFPase revealed that Asp229 in the DFPase is critical to the catalytic reaction [31]. Structural comparison between SMP30/GNL and the squid DFPase showed that Asp204 of SMP30/GNL structurally corresponds to Asp229 in the squid DFPase; Asp204 in SMP30/GNL may function as a catalytic residue of the DFPase activity.

## Discussion

In this study, we determined the crystal structures of mouse SMP30/GNL, which catalyzes formation of the γ-lactone-ring of l-gulonate in the ascorbic acid biosynthetic pathway [9], in substrate-free and substrate/product-analogue complex forms. This is the first determination of a crystal structure of a γ-lactone-forming enzyme in the ascorbic acid biosynthetic pathway. Since human cells have a loss-of-function genetic mutation on gluconolactone oxidase, which is the last enzyme in the ascorbic acid biosynthesis pathway (Figure 1A), human cells cannot synthesize ascorbic acid. Therefore, human SMP30/GNL, the crystal structure of which was determined earlier [11], may have a biological function other than ascorbic acid biosynthesis. Furthermore, our database search showed that this is the first report of a GNL family enzyme in complex with substrate/product-analogue molecules.

The crystal structures of the mouse SMP30/GNL revealed unique characteristics of this enzyme. First, the substrate-binding cavity of mouse SMP30/GNL seems to be designed to recognize various monosaccharide molecules. Polar amino acid residues (Arg101, Asn103, and Glu121) that form hydrogen bonds with hydroxyl groups of a bound molecule are located at one side of the inner surface of the substrate-binding cavity. On the opposite side of the inner surface, there are fewer polar amino-acid residues (Cys16, Ile202, and Tyr219). Due to this asymmetric arrangement of polar residues in the substrate-binding cavity, substrate/product(-analogue) molecules are placed in a specific manner in the cavity. An earlier biochemical investigation reported that a mutation at Asn103 markedly reduced the GNL activity of human SMP30/GNL [11]. Since Asn103 interacts with the hydroxyl groups of bound substrate/product-analogues, the mutation seemed to affect the substrate binding to the active site, resulting in the reduction of GNL activity.

Second, mouse SMP30/GNL has a relatively small substrate-binding cavity. In other related enzymes such as DFPase, Drp35, and PON, the substrate-binding cavity is open to the solvent (**Figure 5**), suggesting that their substrate specificities would be different from that of mouse SMP30/GNL. Furthermore, a structural comparison of mouse and human SMP30/GNL showed that the size of the substrate-binding cavity of mouse SMP30/GNL is significantly smaller than that of the human SMP30/GNL due to the structural difference of their lid loops (**Figure 3A**). The difference of the binding mode of 1,5-AG (**Figure 4B, C**) and the GNL activity between mouse and human SMP30/GNL could be explained by the structural difference of the lid loop.

The relatively small substrate-binding cavity of mouse SMP30/GNL seems to be critical for the γ-lactone-ring formation. The lid loop of mouse SMP30/GNL covers the substrate-binding cavity and seems to restrict the conformation of a bound molecule by interacting with the molecule; the xylitol molecule in the substrate-binding cavity of mouse SMP30/GNL was not able to adopt an extended conformation and adopted a folded conformation instead (**Figure 4E**). Therefore, it is reasonable to predict that l-gulonate also adopts a folded conformation in the substrate-binding cavity of mouse SMP30/GNL. In order to catalyze a ring-forming reaction of l-gulonate, the carbon chain of the substrate should be folded in the reaction.

**Figure 6:**
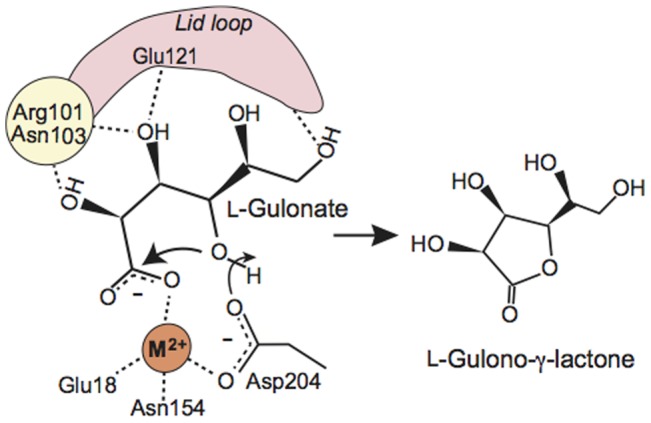
Proposed catalytic reaction mechanism of mouse SMP30/GNL. The divalent metal ion in the active site is labeled as M^2+^.

On the basis of these structural features of mouse SMP30/GNL, the catalytic mechanism of the γ-lactone formation from l-gulonate can be predicted (**Figure 6**). Initially, the carboxylate group of l-gulonate would be coordinated to the divalent metal ion. Then, Arg101, Asn103, and Glu121 seem to interact with the l-gulonate in the substrate-binding cavity, and the l-gulonate seems to bind to the substrate-binding cavity in a folded conformation (**Figure 6**). A manual modeling study suggested that l-gulonate in a folded conformation could be accommodated by the substrate-binding cavity of mouse SMP30/GNL (**Figure S8**). Then, nucleophilic attack of the hydroxyl group at C4 to the C1 atom may lead to the formation of the γ-lactone ring (**Figure 6**). In this step, Asp204 may serve as a catalytic base, which deprotonates OH(4) of the substrate, to facilitate the nucleophilic attack. In this catalytic reaction, hydroxyl groups of the substrate seem to be recognized by Arg101, Asn103, and Glu121, which are located at the one side of the inner surface of the substrate-binding cavity. These interactions seem to properly place the substrate in the active site and induce the substrate binding in a folded conformation.

Our crystal structure analysis of SMP30/GNL showed that a divalent metal ion is located at the active site of SMP30/GNL. The ICP-MS analysis of the purified recombinant SMP30/GNL, which was used for the crystallization, showed that the purified SMP30/GNL contained not only the Ca^2+^ ion but also the Zn^2+^, Mg^2+^, and Mn^2+^ ions. This result suggested that each of the SMP30/GNL molecules in the crystal bound one of these divalent metal ions in the active site. Since the purification conditions of SMP30/GNL (*e.g.*, the concentrations of protein and metal ions in *E. coli* cells and the purification process) must affect the metal ion in the active site of SMP30/GNL, it is very difficult to identify the metal ion in the functional SMP30/GNL in the cell. On the basis of the *K*_d_ values for divalent metal ions, Chakaraborti and Bahnson proposed that cellular conditions might affect the divalent metal ion within the active site of SMP30/GNL [11]. While some groups proposed that SMP30/GNL has multiple cellular functions relevant to the Ca^2+^ ion, it is also possible that other divalent metal ions such as Mg^2+^, Zn^2+^, and Mn^2+^ play a critical role in the cell, particularly its enzymatic functions. Under certain conditions, the Ca^2+^ ion can be bound to the active site of SMP30/GNL [11]. In order to reveal the relationship between metal ions and the biological functions of SMP30/GNL, more biochemical and biological studies are required.

Our structural study of SMP30/GNL has revealed unique structural features of mouse SMP30/GNL, particularly around the substrate-binding cavity. The substrate-binding cavity of mouse SMP30/GNL seems to have suitable structural features for the γ-lactone-forming reaction. Since there are no good methods to measure the enzyme activity of the γ-lactone formation, it is still difficult to enzymatically prove the hypothesis presented in this study. To understand further details of the catalytic reaction of mouse SMP30/GNL, additional biochemical and biological studies will be needed.

## Supporting Information

Figure S1 **Purification and crystallization of mouse and human SMP30/GNL.** (**A, B**) SDS-PAGE of purified mouse (**A**) and human (**B**) SMP30/GNL. (**C, D**) Crystals of mouse (**C**) and human (**D**) SMP30/GNL.
(PDF)

Figure S2 **Molecular weight analysis of mouse and human SMP30/GNL.** LS and RI represent the intensity of static light scattering and the refractive index, respectively. Data points of standard proteins are shown in black circles. Data points for mouse and human SMP30/GNL are shown in blue and red with errors, respectively.
(PDF)

Figure S3 **Ramachandran plots for all determined crystal structures in this study.** (**A**) Mouse SMP30/GNL, (**B**) the mouse SMP30/GNL–1,5-AG complex, (**C**) the mouse SMP30/GNL–d-glucose complex, (**D**) the mouse SMP30/GNL–xylitol complex, (**E**) human SMP30/GNL, and (**F**) the human SMP30/GNL–1,5-AG complex.
(PDF)

Figure S4 **Structural comparison of the lid loops in mouse and human SMP30/GNL in the substrate/product-analogue complex forms.** (**A**) Structural comparison of mouse (purple) and human (blue) SMP30/GNL in complex with 1,5-AG. The lid loop shown in white is that of mouse SMP30/GNL in the substrate-free form. (**B**) SA-omit map for residues in the lid loop of the mouse SMP30/GNL–1,5-AG complex (counter level: 3.0 σ) and (**C**) that of the human SMP30/GNL–1,5-AG complex (counter level: 2.0 σ). (**D**) Structural comparison of mouse SMP30/GNL in the substrate-free (white) and the d-glucose complex (purple) forms. (**E**) SA-omit map of residues in the lid loop of the mouse SMP30/GNL–glucose complex (counter level: 3.0 σ). (**F**) Structural comparison of mouse SMP30/GNL in the substrate-free (white) and the xylitol complex (purple) forms. (**G**) SA-omit map of residues in the lid loop of the mouse SMP30/GNL–xylitol complex (counter level: 3.0 σ). Carbon atoms of 1,5-AG, d-glucose, and xylitol are shown in yellow. Carbon atoms in residues coordinated to the divalent metal ion (orange sphere, labeled as M^2+^) are shown in green. All electron densities are mFo-DFc maps (cyan).
(PDF)

Figure S5 **Sequence comparison of mouse and human SMP30/GNL.** (**A**) The sequence alignment table of mouse and human SMP30/GNL. The residues in the lid loop are indicated by a red box. (**B**) Distribution of the non-conserved residues between mouse and human SMP30/GNL. Non-conserved residues are shown in red.
(PDF)

Figure S6 **SA-omit maps for bound substrate/product-analogues.** (**A**) The mouse SMP30/GNL–1,5-AG complex, (**B**) the human SMP30/GNL–1,5-AG complex, (**C**) the mouse SMP30/GNL–d-glucose complex, and (**D**) the mouse SMP30/GNL–xylitol complex. Carbon atoms in 1,5-AG, d-glucose, and xylitol are shown in yellow. Carbon atoms in residues coordinated to the divalent metal ion (orange sphere, labeled as M^2+^) are shown in green. All electron densities are mFo-DFc maps (contour level: 3 σ).
(PDF)

Figure S7 **B-factor plots of human and mouse SMP30/GNL.** Average B-factors of main chain atoms are plotted for human (**A**) and mouse (**B**) SMP30/GNL. Substrate-free and 1,5-AG complex forms are shown in red and blue lines, respectively. B-factor values of the residues in the lid loop are plotted with thick lines.
(PDF)

Figure S8 **Model structure of the mouse SMP30/GNL–l-gulonate complex.** The model was manually prepared on the basis of the crystal structure of the mouse SMP30/GNL–xylitol complex. The model structure was structurally idealized using Refmac5 [32]. l-Gulonate can be accommodated in the substrate-binding cavity with a folded-conformation. Carbon atoms in l-gulonate are shown in yellow. Carbon atoms in residues coordinated to the divalent metal ion (orange sphere, labeled as M^2+^) are shown in green. Residues in the lid loop are shown in purple. The thin white molecule is a xylitol molecule superposed on the model structure. The l-gulonate occupies nearly the same position as that of xylitol in this model.
(PDF)

Table S1 **GNL activity of crude extract from** ***E. coli*** **with and without SMP30/GNL gene.**
(PDF)

Table S2 **Effect of crude extract on the GNL activity of SMP30/GNL.**
(PDF)

Table S3 **Divalent metal ion contents in the purified human and mouse SMP30/GNL.**
(PDF)

Table S4 **Rms deviations (Å) from the least-squares fittings of mouse and human SMP30/GNL.**
(PDF)

Table S5 **Coordination distances and temperature factors of the divalent metal ion (M^2+^) and its ligand atoms in the substrate free form.**
(PDF)

Table S6 **Coordination distances and temperature factors of the divalent metal ion (M^2+^) and its ligand atoms in the 1,5-AG complex.**
(PDF)

Table S7 **Coordination distances and temperature factors of the divalent metal ion (M^2+^) and its ligand atoms in the glucose and xylitol complexes.**
(PDF)

Table S8 **RMS deviations from SSM fitting between mouse SMP30/GNL in the substrate-free form and other related structures.**
(PDF)
