# Supplementary figures and images for: Structural Basis of the γ-Lactone-Ring Formation in Ascorbic Acid Biosynthesis by the Senescence Marker Protein-30/Gluconolactonase
Source: PLoS One. 2013 Jan 22;8(1):e53706. doi: 10.1371/journal.pone.0053706 (PMC3551927; doi:10.1371/journal.pone.0053706)

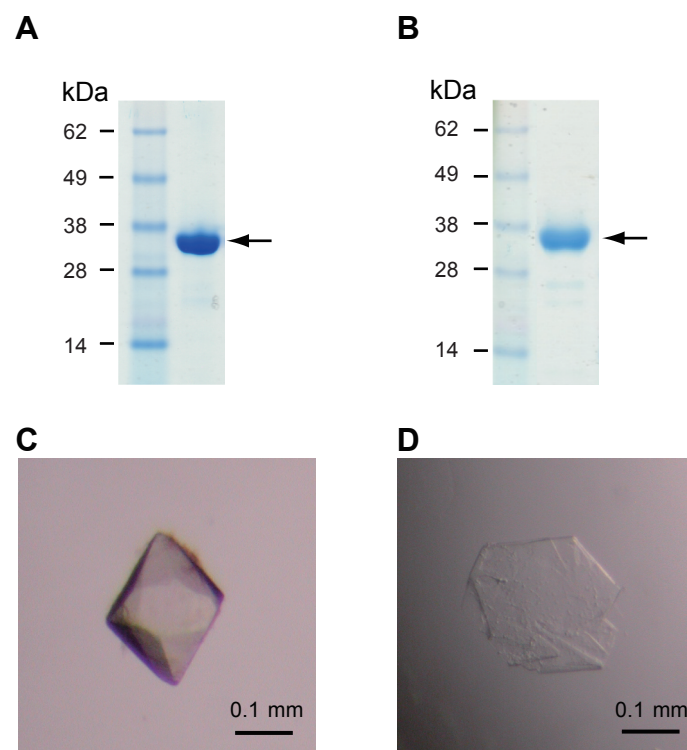

Figure S1

Supplement: Figure S1 — Purification and crystallization of mouse and human SMP30/GNL. (A, B) SDS-PAGE of purified mouse (A) and human (B) SMP30/GNL. (C, D) Crystals of mouse (C) and human (D) SMP30/GNL. (PDF) [file pone.0053706.s001.pdf]

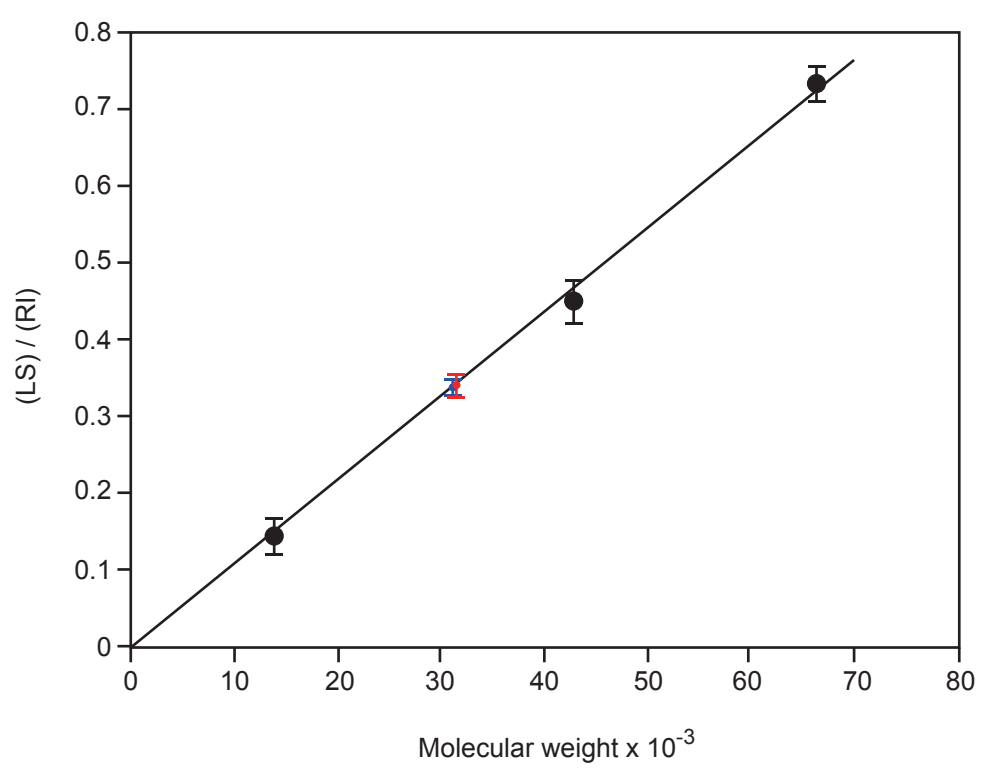

Figure S2

Supplement: Figure S2 — Molecular weight analysis of mouse and human SMP30/GNL. LS and RI represent the intensity of static light scattering and the refractive index, respectively. Data points of standard proteins are shown in black circles. Data points for mouse and human SMP30/GNL are shown in blue and red with errors, respectively. (PDF) [file pone.0053706.s002.pdf]

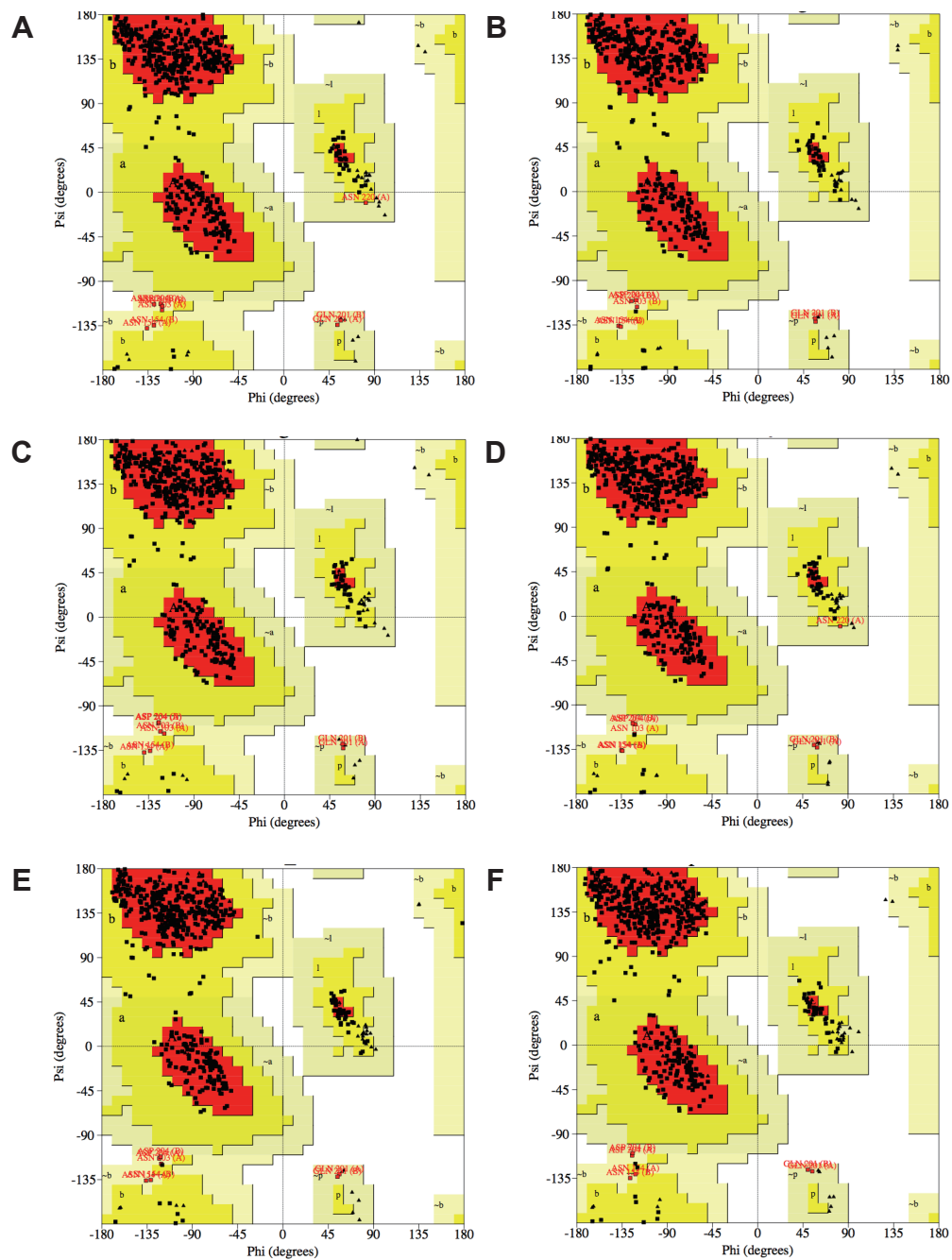

Figure S3

Supplement: Figure S3 — Ramachandran plots for all determined crystal structures in this study. (A) Mouse SMP30/GNL, (B) the mouse SMP30/GNL–1,5-AG complex, (C) the mouse SMP30/GNL–d-glucose complex, (D) the mouse SMP30/GNL–xylitol complex, (E) human SMP30/GNL, and (F) the human SMP30/GNL–1,5-AG complex. (PDF) [file pone.0053706.s003.pdf]

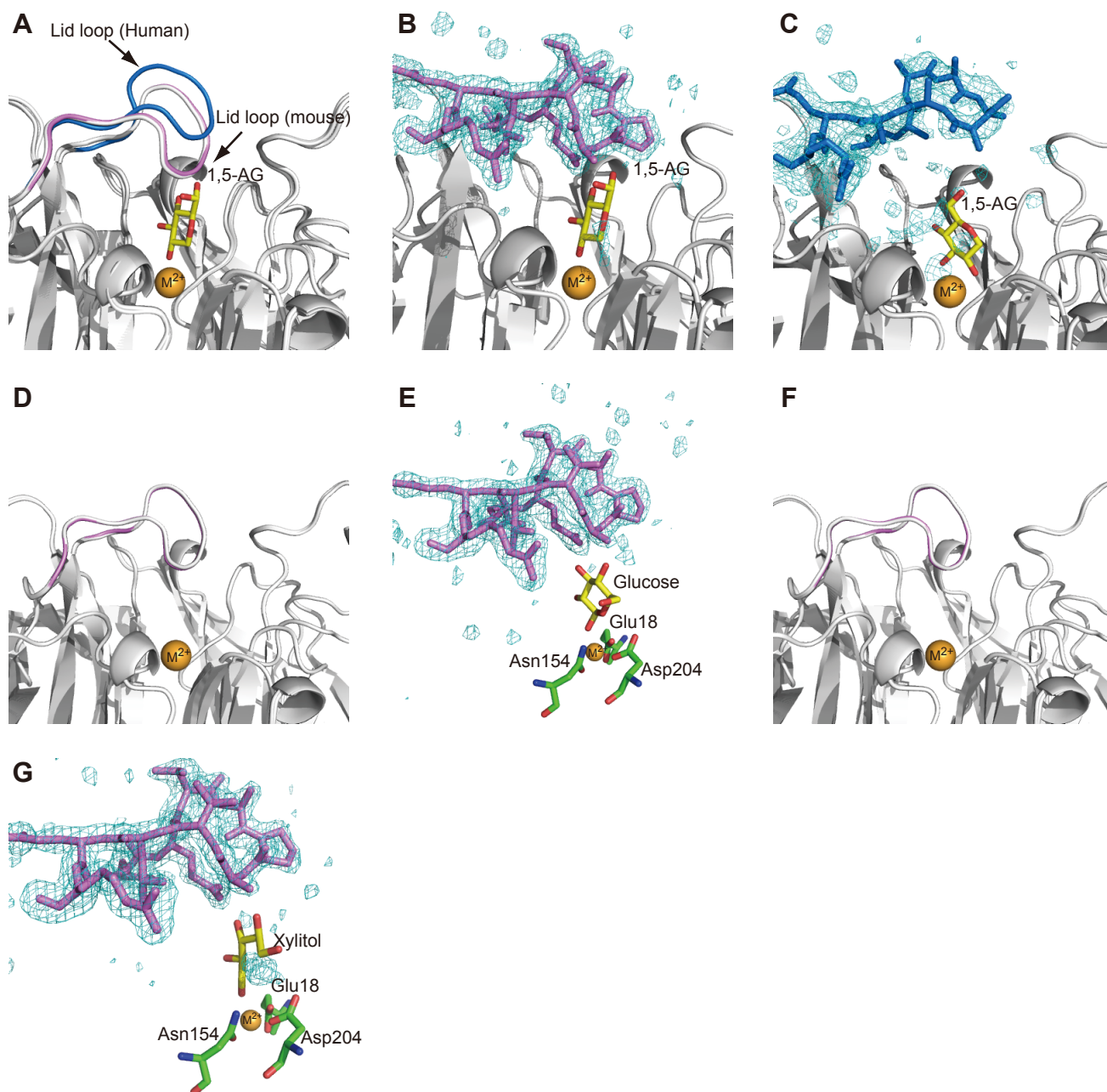

Figure S4

Supplement: Figure S4 — Structural comparison of the lid loops in mouse and human SMP30/GNL in the substrate/product-analogue complex forms. (A) Structural comparison of mouse (purple) and human (blue) SMP30/GNL in complex with 1,5-AG. The lid loop shown in white is that of mouse SMP30/GNL in the substrate-free form. (B) SA-omit map for residues in the lid loop of the mouse SMP30/GNL–1,5-AG complex (counter level: 3.0 σ) and (C) that of the human SMP30/GNL–1,5-AG complex (counter level: 2.0 σ). (D) Structural comparison of mouse SMP30/GNL in the substrate-free (white) and the d-glucose complex (purple) forms. (E) SA-omit map of residues in the lid loop of the mouse SMP30/GNL–glucose complex (counter level: 3.0 σ). (F) Structural comparison of mouse SMP30/GNL in the substrate-free (white) and the xylitol complex (purple) forms. (G) SA-omit map of residues in the lid loop of the mouse SMP30/GNL–xylitol complex (counter level: 3.0 σ). Carbon atoms of 1,5-AG, d-glucose, and xylitol are shown in yellow. Carbon atoms in residues coordinated to the divalent metal ion (orange sphere, labeled as M2+) are shown in green. All electron densities are mFo-DFc maps (cyan). (PDF) [file pone.0053706.s004.pdf]

**A**

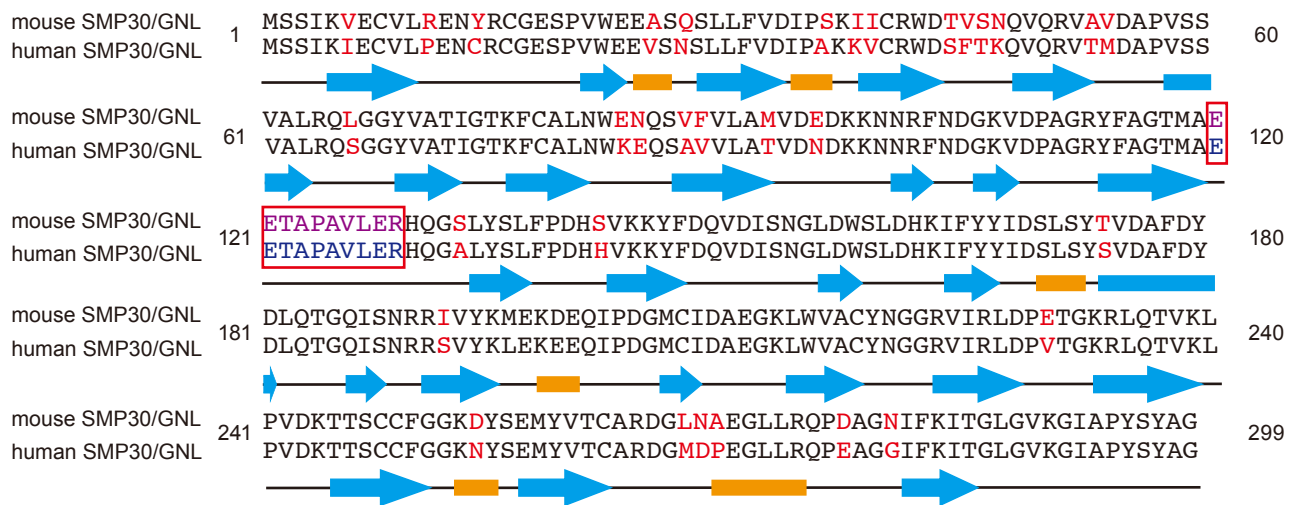

**B**

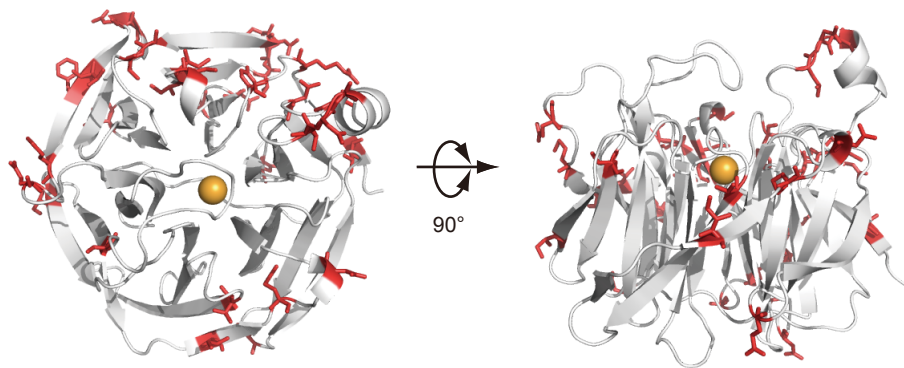

Figure S5

Supplement: Figure S5 — Sequence comparison of mouse and human SMP30/GNL. (A) The sequence alignment table of mouse and human SMP30/GNL. The residues in the lid loop are indicated by a red box. (B) Distribution of the non-conserved residues between mouse and human SMP30/GNL. Non-conserved residues are shown in red. (PDF) [file pone.0053706.s005.pdf]

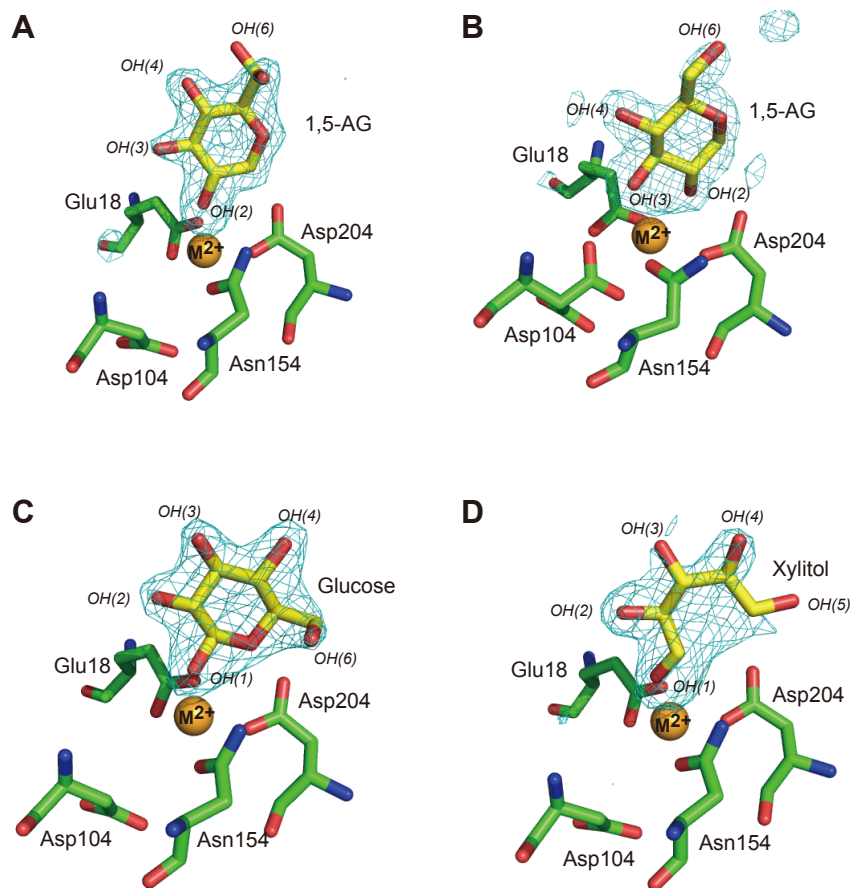

Figure S6

Supplement: Figure S6 — SA-omit maps for bound substrate/product-analogues. (A) The mouse SMP30/GNL–1,5-AG complex, (B) the human SMP30/GNL–1,5-AG complex, (C) the mouse SMP30/GNL–d-glucose complex, and (D) the mouse SMP30/GNL–xylitol complex. Carbon atoms in 1,5-AG, d-glucose, and xylitol are shown in yellow. Carbon atoms in residues coordinated to the divalent metal ion (orange sphere, labeled as M2+) are shown in green. All electron densities are mFo-DFc maps (contour level: 3 σ). (PDF) [file pone.0053706.s006.pdf]

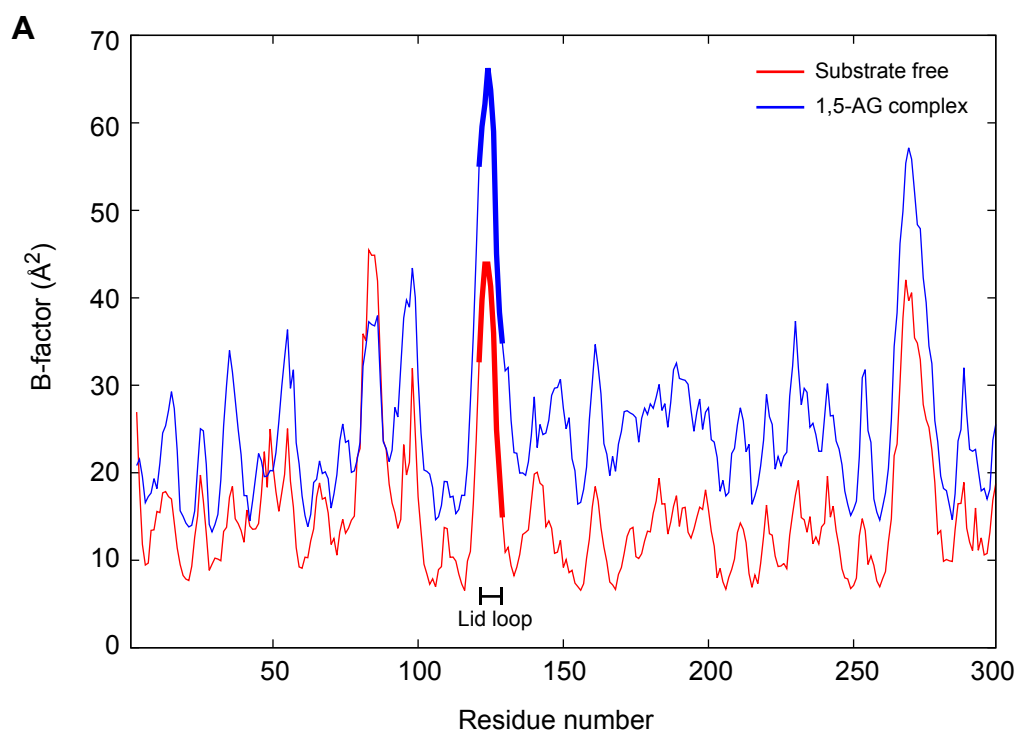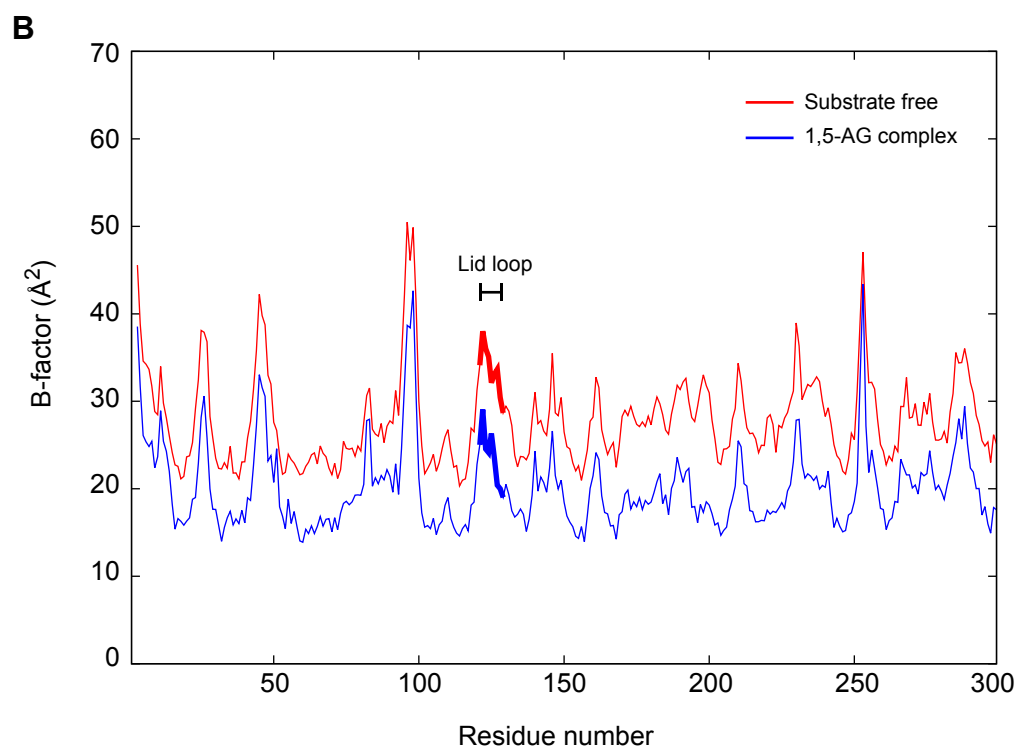

Figure S7

Supplement: Figure S7 — B-factor plots of human and mouse SMP30/GNL. Average B-factors of main chain atoms are plotted for human (A) and mouse (B) SMP30/GNL. Substrate-free and 1,5-AG complex forms are shown in red and blue lines, respectively. B-factor values of the residues in the lid loop are plotted with thick lines. (PDF) [file pone.0053706.s007.pdf]

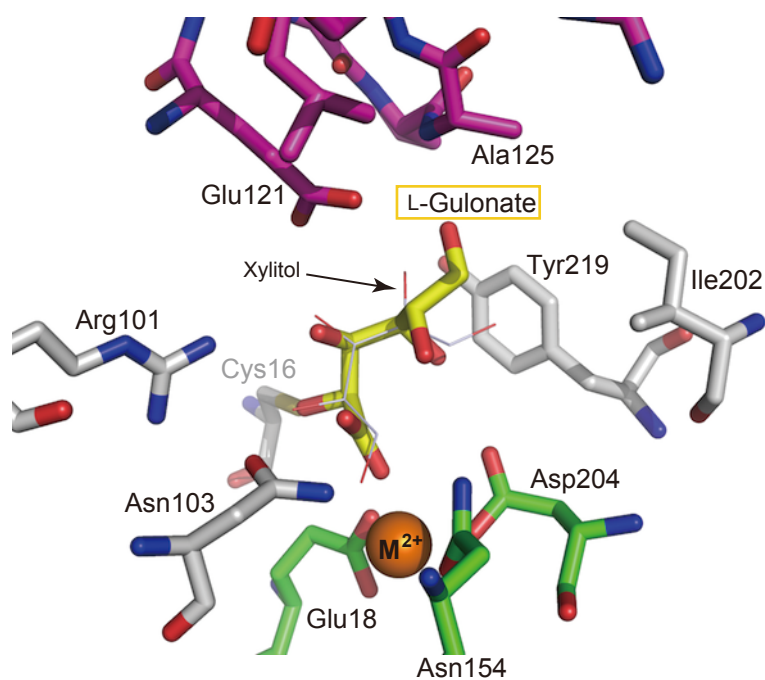

Figure S8

Supplement: Figure S8 — Model structure of the mouse SMP30/GNL–l-gulonate complex. The model was manually prepared on the basis of the crystal structure of the mouse SMP30/GNL–xylitol complex. The model structure was structurally idealized using Refmac5 [32]. l-Gulonate can be accommodated in the substrate-binding cavity with a folded-conformation. Carbon atoms in l-gulonate are shown in yellow. Carbon atoms in residues coordinated to the divalent metal ion (orange sphere, labeled as M2+) are shown in green. Residues in the lid loop are shown in purple. The thin white molecule is a xylitol molecule superposed on the model structure. The l-gulonate occupies nearly the same position as that of xylitol in this model. (PDF) [file pone.0053706.s008.pdf]
