# Supplementary material for: Structural Basis of the γ-Lactone-Ring Formation in Ascorbic Acid Biosynthesis by the Senescence Marker Protein-30/Gluconolactonase
Source: PLoS One. 2013 Jan 22;8(1):e53706. doi: 10.1371/journal.pone.0053706 (PMC3551927; doi:10.1371/journal.pone.0053706)
Supplement: Table S1 — GNL activity of crude extract from E. coli with and without SMP30/GNL gene. (PDF) [file pone.0053706.s009.pdf]

Table S1. GNL activity of crude extract from *E. coli* with and without SMP30/GNL gene

***E. coli*/expression vector**	**GNL activity (μmol/min/mg)**
BL21(DE3)-Rossetta2/pLysS [pET21b(+)]	N.D.*
BL21(DE3)-Rossetta2/pLysS [pET21b(+)/human SMP30/GNL]	25 ± 0.3
BL21(DE3) [pET21b(+)]	N.D.
BL21(DE3) [pET21b(+)/mouse SMP30/GNL]	8.8 ± 0.4

N.D.: No GNL activity was detected.
