# Supplementary material for: Structural Basis of the γ-Lactone-Ring Formation in Ascorbic Acid Biosynthesis by the Senescence Marker Protein-30/Gluconolactonase
Source: PLoS One. 2013 Jan 22;8(1):e53706. doi: 10.1371/journal.pone.0053706 (PMC3551927; doi:10.1371/journal.pone.0053706)
Supplement: Table S2 — Effect of crude extract on the GNL activity of SMP30/GNL. (PDF) [file pone.0053706.s010.pdf]

Table S2. Effect of crude extract on the GNL activity of SMP30/GNL

	Crude extract* (μg†)			
	0.1	1	5	10
	GNL activity (μmol/min/mg)			
Human SMP30/GNL	681 ± 45	681 ± 36	692 ± 45	654 ± 49
Mouse SMP30/GNL	723 ± 65	667 ± 40	692 ± 52	739 ± 51

* Crude extract was prepared from *E. coli* without SMP30/GNL gene.

† Total amount of proteins in the crude extract was estimated by the Bradford method using bovine serum albumin as a standard.
