# Supplementary material for: Structural Basis of the γ-Lactone-Ring Formation in Ascorbic Acid Biosynthesis by the Senescence Marker Protein-30/Gluconolactonase
Source: PLoS One. 2013 Jan 22;8(1):e53706. doi: 10.1371/journal.pone.0053706 (PMC3551927; doi:10.1371/journal.pone.0053706)
Supplement: Table S3 — Divalent metal ion contents in the purified human and mouse SMP30/GNL. (PDF) [file pone.0053706.s011.pdf]

Table S3. Divalent metal ion contents in the purified human and mouse SMP30/GNL

	Mouse[*1]			Human[*1]		
Sample[*2]	Purified	Dialyzed	Chelated	Purified	Dialyzed	Chelated
Protein conc. (mg/ml)	1.2	0.5	0.8	1.2	1.2	1.2
Concentration (μM) [*3]						
Mg^{2+}	7.0	N.D.	0.29	5.4	N.D.	N.D.
Ca^{2+}	18.0	0.75	0.97	17.0	1.85	0.87
Mn^{2+}	0.35	0.04	N.D. [*4]	0.16	0.27	N.D.
Zn^{2+}	0.55	0.55	N.D.	1.10	N.D.	N.D.
Metal ions per purified SMP30/GNL (%) [*5]						
Mg^{2+}	19	N.D.	1	15	N.D.	N.D.
Ca^{2+}	51	5	4	46	3.9	1.9
Mn^{2+}	1.0	0.2	N.D.	0.5	0.6	N.D.
Zn^{2+}	1.5	3.7	N.D.	3.0	N.D.	N.D.

*1 Mouse: Mouse SMP30/GNL, Human: Human SMP30/GNL

*2 Purified: purified protein, Dialyzed: dialyzed protein without chelating reagents., Chelated: dialyzed protein with chelating reagents.

*3 Concentrations of the divalent metal ions in the sample solutions analyzed by ICP-MS.

*4 N.D. indicates that the concentration of divalent metal ions was less than10 ppb, which was the lower limit of detection.

*5 Percentages of divalent metal ions calculated on a molar basis. Data are expressed as μ mol of divalent metal ions per μ mol of purified mouse or human SMP30/GNL.
