# Supplementary material for: Structural Basis of the γ-Lactone-Ring Formation in Ascorbic Acid Biosynthesis by the Senescence Marker Protein-30/Gluconolactonase
Source: PLoS One. 2013 Jan 22;8(1):e53706. doi: 10.1371/journal.pone.0053706 (PMC3551927; doi:10.1371/journal.pone.0053706)
Supplement: Table S4 — Rms deviations (Å) from the least-squares fittings of mouse and human SMP30/GNL. (PDF) [file pone.0053706.s012.pdf]

Table S4. Rms deviations (Å) from the least-squares fittings of mouse and human SMP30/GNL.

	Mouse A*	Mouse B*	Human A*	Human B*
Mouse A				
Mouse B	0.34			
Human A	0.62	0.59		
Human B	0.68	0.64	0.42	

* Mouse A: mouse SMP30/GNL chain A; Mouse B: mouse SMP30/GNL chain B; Human A: human SMP30/GNL chain A; Human B: human SMP30/GNL chain B. Human SMP30/GNL coordinates determined in this study were used.

Rms deviations (Å) were calculated by Lsqkab in the CCP4 program suite [24]. Least-squares fittings were performed with 297 corresponding Cα atoms.
