# Supplementary material for: Structural Basis of the γ-Lactone-Ring Formation in Ascorbic Acid Biosynthesis by the Senescence Marker Protein-30/Gluconolactonase
Source: PLoS One. 2013 Jan 22;8(1):e53706. doi: 10.1371/journal.pone.0053706 (PMC3551927; doi:10.1371/journal.pone.0053706)
Supplement: Table S5 — Coordination distances and temperature factors of the divalent metal ion (M2+) and its ligand atoms in the substrate free form. (PDF) [file pone.0053706.s013.pdf]

Table S5. Coordination distances and temperature factors of the divalent metal ion (M^{2+}) and its ligand atoms in the substrate free form

	Substrate free (mouse)		Substrate free (human)[*1]	
	Chain A	Chain B	Chain A	Chain B
		Distance (Å)		
M^{2+}-Glu18	2.2	2.3	2.3	2.3
M^{2+}-Asn154	2.4	2.2	2.3	2.2
M^{2+}-Asp204	2.2	2.3	2.3	2.2
M^{2+}-Wat1	2.4	-	2.3	2.3
M^{2+}-Wat2	2.3	2.4	2.4	2.4
M^{2+}-Wat3	2.1	2.3	2.3	2.4
		B-factor ($Å^2$)		
M^{2+}	32.6	47.4	11.0	10.8
Glu18	36.0	37 .6	18.5	23.7
Asn154	29.4	36.8	11.1	13.7
Asp204	30.4	29.8	9.6	11.5
Wat1	34.3	-	15.8	16.9
Wat2	31.0	44.2	16.6	16.4
Wat3	26.8	35.4	11.6	14.2

*1 Human SMP30/GNL coordinates determined in this study were used.
