# Supplementary material for: Structural Basis of the γ-Lactone-Ring Formation in Ascorbic Acid Biosynthesis by the Senescence Marker Protein-30/Gluconolactonase
Source: PLoS One. 2013 Jan 22;8(1):e53706. doi: 10.1371/journal.pone.0053706 (PMC3551927; doi:10.1371/journal.pone.0053706)
Supplement: Table S6 — Coordination distances and temperature factors of the divalent metal ion (M2+) and its ligand atoms in the 1,5-AG complex. (PDF) [file pone.0053706.s014.pdf]

Table S6. Coordination distances and temperature factors of the divalent metal ion (M^{2+}) and its ligand atoms in the 1,5-AG complex

	1,5-AG (mouse)		1,5-AG (human)	
	Chain A	Chain B	Chain A	Chain B
	Distance (Å)			
M^{2+}-Glu18	2.1	2.2	2.3	2.3
M^{2+}-Asn154	2.1	2.2	2.1	2.1
M^{2+}-Asp204	2.1	2.3	2.3	2.3
M^{2+}-Asp104	-	-	2.4	2.4
M^{2+}-$OH(2)_{1,5\text{-}AG}$ *2	2.2	2.1	2.4	2.4
M^{2+}-$OH(3)_{1,5\text{-}AG}$ *2	-	-	2.5	2.4
M^{2+}-Wat1	2.2	2.2	2.3	2.3
M^{2+}-Wat2	2.5	-	-	-
	B-factor ($Å^2$)			
M^{2+}	16.0	24.8	21.8	19.0
Glu18	23.7	32.6	27.6	29.3
Asn154	17.0	21.0	25.1	25.5
Asp204	23.4	26.4	21.2	18.8
Asp104	-	-	21.4	19.6
$OH(2)_{1,5\text{-}AG}$ *1	21.3	22.8	30.7	28.0
$OH(3)_{1,5\text{-}AG}$ *1	-	-	33.5	24.6
Wat1	25.7	33.3	30.6	27.3
Wat2	34.1	-	-	-

*2 $OH(2)_{1,5\text{-}AG}$ and $OH(3)_{1,5\text{-}AG}$ represent a hydroxyl group at C2 and C3 of 1,5-AG, respectively.
