# Supplementary material for: Structural Basis of the γ-Lactone-Ring Formation in Ascorbic Acid Biosynthesis by the Senescence Marker Protein-30/Gluconolactonase
Source: PLoS One. 2013 Jan 22;8(1):e53706. doi: 10.1371/journal.pone.0053706 (PMC3551927; doi:10.1371/journal.pone.0053706)
Supplement: Table S7 — Coordination distances and temperature factors of the divalent metal ion (M2+) and its ligand atoms in the glucose and xylitol complexes. (PDF) [file pone.0053706.s015.pdf]

Table S7. Coordination distances and temperature factors of the divalent metal ion (M^{2+}) and its ligand atoms in the glucose and xylitol complexes

	D-Glucose complex (mouse)		Xylitol complex (mouse)	
	Chain A	Chain B	Chain A	Chain B
	Distance (Å)			
M^{2+}-Glu18	2.2	2.1	2.2	-
M^{2+}-Asn154	2.3	2.4	2.2	-
M^{2+}-Asp204	2.1	2.2	2.1	-
M^{2+}-$OH(1)_{Glc}$ *3	2.0	2.0	-	-
M^{2+}-$OH(1)_{Xyl}$ *4	-	-	1.9	-
M^{2+}-Wat1	2.3	2.2	2.1	-
M^{2+}-Wat2	2.6	-	2.4	-
	B-factor ($Å^2$)			
M^{2+}	40.9	35.4	19.5	-
Glu18	33.7	40.4	31.4	-
Asn154	24.9	28.0	24.0	-
Asp204	32.7	34.7	26.1	-
$OH(1)_{Glc}$ *3	31.8	29.3	-	-
$OH(1)_{Xyl}$ *4	-	-	36.9	-
Wat1	32.1	33.4	28.1	-
Wat2	37.6	-	31.7	-

*3 $OH(1)_{Glc}$ represents a hydroxyl group at C1 of D-glucose.
*4 $OH(1)_{Xyl}$ represents a hydroxyl group at C1 of xylitol.
