# Supplementary material for: Structural Basis of the γ-Lactone-Ring Formation in Ascorbic Acid Biosynthesis by the Senescence Marker Protein-30/Gluconolactonase
Source: PLoS One. 2013 Jan 22;8(1):e53706. doi: 10.1371/journal.pone.0053706 (PMC3551927; doi:10.1371/journal.pone.0053706)
Supplement: Table S8 — RMS deviations from SSM fitting between mouse SMP30/GNL in the substrate-free form and other related structures. (PDF) [file pone.0053706.s016.pdf]

Table S8. RMS deviations from SSM fitting between mouse SMP30/GNL in the substrate-free form and other related structures.

Enzyme	rmsd (Å)	Alignment length
mouse SMP30/GNL-1,5AG complex	0.14	297
DFPase (1PJX) *	1.8	254
Drp35 (2DG1) *	2.1	250
PON (1V04)*	2.9	234

* PDB ID is given in parentheses.
